# Augmented Reality Learning Environment for Basic Life Support and Defibrillation Training: Usability Study

**DOI:** 10.2196/14910

**Published:** 2020-05-12

**Authors:** Pier Luigi Ingrassia, Giulia Mormando, Eleonora Giudici, Francesco Strada, Fabio Carfagna, Fabrizio Lamberti, Andrea Bottino

**Affiliations:** 1 SIMNOVA - Centro di Simulazione in Medicina e Professioni Sanitarie Università del Piemonte Orientale Novara Italy; 2 Department of Medicine Università di Padova Padova Italy; 3 School of Medicine Università Piemonte Orientale Novara Italy; 4 Department of Control and Computer Engineering Politecnico di Torino Torino Italy

**Keywords:** augmented reality, cardiopulmonary resuscitation, automated external defibrillators

## Abstract

**Background:**

Basic life support (BLS) is crucial in the emergency response system, as sudden cardiac arrest is still a major cause of death worldwide. Unfortunately, only a minority of victims receive cardiopulmonary resuscitation (CPR) from bystanders. In this context, training could be helpful to save more lives, and technology-enhanced BLS simulation is one possible solution.

**Objective:**

The aim of this study is to assess the feasibility and acceptability of our augmented reality (AR) prototype as a tool for BLS training.

**Methods:**

Holo-BLSD is an AR self-instruction training system, in which a standard CPR manikin is “augmented” with an interactive virtual environment that reproduces realistic scenarios. Learners can use natural gestures, body movements, and spoken commands to perform their tasks, with virtual 3D objects anchored to the manikin and the environment. During the experience, users were trained to use the device while being guided through an emergency simulation and, at the end, were asked to complete a survey to assess the feasibility and acceptability of the proposed tool (5-point Likert scale; 1=Strongly Disagree, 5=Strongly Agree).

**Results:**

The system was rated easy to use (mean 4.00, SD 0.94), and the trainees stated that most people would learn to use it very quickly (mean 4.00, SD 0.89). Voice (mean 4.48, SD 0.87), gaze (mean 4.12, SD 0.97), and gesture interaction (mean 3.84, SD 1.14) were judged positively, although some hand gesture recognition errors reduced the feeling of having the right level of control over the system (mean 3.40, SD 1.04).

**Conclusions:**

We found the Holo-BLSD system to be a feasible and acceptable tool for AR BLS training.

## Introduction

Sudden cardiac arrest is a major cause of death in adults in developed countries [1]. As such, basic life support (BLS) is a fundamental aspect of the emergency response system. Survival rates are higher when bystanders are able to deliver early cardiopulmonary resuscitation (CPR) and defibrillation [2]. Unfortunately, although laypeople and health care providers are increasingly trained, only a minority of cardiac arrest survivors receive bystander CPR. Therefore, despite major gaps that still exist in the delivery of optimal care [3], training plays a pivotal role in saving lives [4].

The standard approach to BLS training of laypeople involves classroom-based courses consisting of skill demonstrations, hands-on practice, and lectures given by a certified trainer [5]. Simulation with manikins allows trainees to acquire and practice skills without the risk of harming the patient [6]. Debriefing provides an important learning moment in simulation sessions, as it gives participants the opportunity to critically reflect on decisions and actions performed and to learn from mistakes [7]. Although it is still a matter of debate how simulation realism influences learning outcomes [4], the importance of elements of stress and cognitive load in education has been proven, and they should be factored into the instructional design [8]. Self-directed learning systems are an effective alternative to the standard approach, which, according to multiple studies reported by the American Heart Association guidelines, show no statistical difference in learning outcomes compared with instructor-led courses [5].

In the context of self-instruction approaches, emerging technologies allow people to build new cognitive structures [7], and technology-enhanced simulation offers new models for training, which are associated with better knowledge, improved skills acquisition, and a moderate effect on patient outcomes [9]. One such emerging technology is augmented reality (AR), which enhances the user’s perception by overlaying virtual objects (or “holograms”) on the real-world environment [10]. AR is being applied across various disciplines in health care education including anatomy classes and surgical training [11-13]. In their integrative review, Zhu et al [14] reported that AR could improve health care education by reducing failure rates and improving accuracy.

In this study, we investigated the use of AR as an innovative technology for BLS training. The aim of this study is to assess feasibility and acceptability of Holo-BLSD, our AR prototype tool for CPR training.

## Methods

### Development

Holo-BLSD was developed jointly by the SIMNOVA simulation center (Novara, Italy) and the Department of Computer Engineering of Politecnico di Torino, in collaboration with Logosnet’s e-REAL Immersive Simulation Labs in Lugano, Switzerland. The app uses Microsoft’s HoloLens device, a wearable headset for AR experiences, and meets the recent American Heart Association guidelines [2]. Holo-BLSD can be used in different real environments, and its contents can be adapted to where the system is being used: virtual elements can be placed and anchored in the desired position.

Since the majority of potential users are likely to have little or no experience with AR apps or the HoloLens, Holo-BLSD provides a specific training session to help users get acquainted with the system. During interaction training, all interaction types are introduced individually with detailed instructions that include vocal and visual clues.

**Figure 1:**
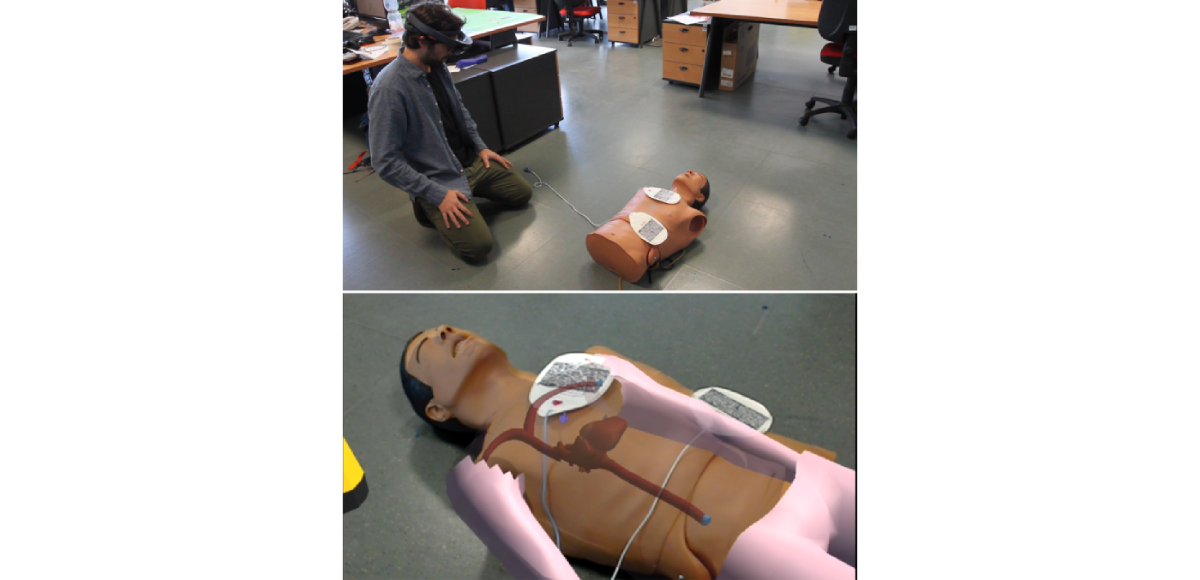
A trainee using the HoloLens device (above) and AR displayed in the trainee’s field of view (below).

After the interaction training, users can begin the BLS training. Holo-BLSD guides users step-by-step through the resuscitation procedure of an adult experiencing cardiac arrest. The simulated activities include the following: scene safety, in which any potential hazard should be removed; responsiveness check, performed by shaking and calling the victim; activation of local emergency medical services by using a public phone booth and interacting with a simulated operator; automated external defibrillator (AED) retrieval by asking a witness to find an AED; CPR, using a real manikin torso and superimposing a person’s full body using AR (Figure 1); use of AED, once available, by directing pad placement and delivery of shock, if recommended. All generated data are logged, and a feedback sheet can be generated, allowing trainees and instructors to intuitively identify errors and strengths of the performance, thus supporting debriefing sessions and enabling the creation of a library of training events.

Participants were recruited on a voluntary basis during a national simulation-based training event for medical residents [15]. The selection of subjects represents a convenience sample, and medical background was not a required inclusion criterion. Experiments took place at the SIMNOVA Simulation Center in Novara, Italy.

The experience was divided into three phases, in which volunteers (1) learned how to use the device through the interaction training session, (2) performed a BLS simulation using the Holo-BLSD app and a standard torso manikin, and (3) filled out the evaluation questionnaire.

### Software Evaluation

Participants completed a survey of 61 questions divided across six categories (user input, system output, system usability, fidelity of simulation, immersivity, likeability), and graded on a scale from 1 (strongly disagree) to 5 (strongly agree). The complete questionnaire can be found in Multimedia Appendix 1.

The results are presented with relative percentages for each of the five possible grades and summarized as mean and standard deviation. Given the explorative nature of the study, we did not plan any statistical inference tests.

### Feasibility

Feasibility was assessed using the user input scale (interaction with gaze and gestures); the system output scale (quality of the display and sound); and the system usability scale [16], which is a standardized tool to evaluate interfaces based on the ISO 9241-400 guidance on ergonomic factors.

### Acceptability

The remaining scales of the survey (fidelity, immersion, and likeability) were designed to assess acceptability.

### Ethics and Statistical Analysis

Written informed consent was obtained from all participants, and the study results were presented in aggregate with no identifiers. The study was conducted in accordance with the principles of the Declaration of Helsinki. Descriptive statistics were calculated using Microsoft Excel (Version 2003, Microsoft Corporation).

## Results

This study involved 26 participants whose characteristics are summarized in Table 1.

### Feasibility

Responses to the feasibility scale are shown in Multimedia Appendices 2 and 3 and aggregated data for the system usability scale are presented in Table 2. The cognitive load required to operate the HoloLens was minimal (mean 1.77, SD 0.86) and no high physical effort was required (mean 1.19, SD 0.40). Users felt confident using the software (mean 3.62, SD 1.06). Voice (mean 4.48, SD 0.87), gaze (mean 4.12, SD 0.97), and gesture interaction (mean 3.84, SD 1.14) were evaluated positively. Words and symbols (mean 4.70, SD 0.56) and audio instructions (mean 4.43, SD 0.79) were easy to understand. Users were most critical of the quality of the display (mean 2.45, SD 1.47), although they rated it as appropriate for the function (mean 3.62, SD 1.17). Finally, users rated the system as easy to use (mean 4.00, SD 0.94).

### Acceptability

Responses to the acceptability scale are summarized in Multimedia Appendices 4-6. The sensorial information provided by the AR gave participants the impression of physically being in the scenario (mean 3.52, SD 0.95). Users reported that the experience was pleasant (mean 4.13, SD 0.81) and enjoyable (mean 4.65, SD 0.57) and that the virtual contents were realistic (mean 3.74, SD 1.05). Users judged the system as capable of providing real benefit as a training tool (mean 4.22, SD 0.67).

**Table 1:** Respondents’ demographics and characteristics^a^.

Characteristic			Participants, n (%)
**Gender**			
	Male		16 (62)
	Female		10 (38)
**Age group (years)**			
	20-29		15 (58)
	30-39		9 (35)
	40-49		0 (0)
	50-59		2 (8)
**Practice type**			
	Resident		19 (73)
	Physician		3 (12)
	Nurse		1 (4)
	**Other**		
		Space system engineer	1 (4)
		Designer	1 (4)
		Secretary	1 (4)
**Specialty^b^**			
	Emergency medicine		7 (32)
	Anesthesiology		6 (27)
	General surgery		2 (9)
	Internal medicine		1 (5)
	Pediatrics		1 (5)
	Cardiology		1 (5)
	Not specified		4 (18)

^a^Percentages may not add up to 100 due to rounding.

^b^Only applicable to residents and physicians.

**Table 2:** Aggregated data for the system usability scale.

	Question	Strongly Disagree (%)	Disagree (%)	Neither (%)	Agree (%)	Strongly Agree (%)	Mean (SD)
**System Usability Scale (SUS)**							
	I think that I would like to use this system frequently	0.0	11.5	23.1	38.5	26.9	3.81 (0.98)
	I found the system unnecessarily complex	48.0	36.0	12.0	4.0	0.0	1.72 (0.84)
	I thought the system was easy to use	0.0	7.7	19.2	38.5	34.6	4.00 (0.94)
	I think that I would need the support of a technical person to be able to use this system	15.4	19.2	30.8	23.1	11.5	2.96 (1.25)
	I found the various functions in this system were well integrated	0.0	3.8	30.8	42.3	23.1	3.85 (0.83)
	I thought there was too much inconsistency in this system	26.9	34.6	23.1	11.5	3.8	2.31 (1.12)
	I would imagine that most people would learn to use this system very quickly	0.0	3.8	26.9	34.6	34.6	4.00 (0.89)
	I found the system very cumbersome to use	32.0	24.0	28.0	8.0	8.0	2.36 (1.25)
	I felt very confident using the system	0.0	23.1	11.5	46.2	19.2	3.62 (1.06)
	I needed to learn a lot of things before I could get going with this system	57.7	26.9	11.5	0.0	3.8	1.65 (0.98)
**ISO 9241-400**							
	The HoloLens device is too bulky or too heavy	19.2	42.3	3.8	26.9	7.7	2.62 (1.30)
	The mental effort (concentration) required to operate the device was very high	46.2	34.6	15.4	3.8	0.0	1.77 (0.86)
	The physical effort required to operate the device was very high	80.8	19.2	0.0	0.0	0.0	1.19 (0.40)
	Arm and hands/fingers fatigue was very high	76.9	19.2	3.8	0.0	0.0	1.27 (0.53)
	Eye fatigue was very high	42.3	23.1	19.2	7.7	7.7	2.15 (1.29)
	Head fatigue was very high	38.5	38.5	15.4	0.0	7.7	2.00 (1.13)
	I would be comfortable using the device for long time	15.4	15.4	30.8	34.6	3.8	2.96 (1.15)

## Discussion

### Principal Findings

Experts are paying increased attention to the realism and scenario design of CPR training. The physical features of manikins and simulators by themselves are insufficient in suspending learners’ disbelief and positively influencing learning outcomes [4]. Despite ongoing advances in resuscitation science, cardiac arrest survival rates remain suboptimal and the educational efficiency of caregivers is still critical, as highlighted in 2003 [17] and 2018 [3]. Education facilitated through technology has been identified as a strategy to improve the effectiveness of BLS training. We developed the Holo-BLSD app using Microsoft’s HoloLens technology as an AR self-instruction learning environment for training and assessment of CPR and AED use, by using high definition holograms to immerge trainees in realistic scenarios, and a standard low-cost torso manikin to deliver tactile feedback. In this pilot study, we measured the feasibility and acceptability of our first prototype of the training app.

An excessive cognitive load may impair participants’ perceptions and performance, decreasing attention and problem-solving skills [6], and physical effort and fatigue may reduce user enjoyment of the experience. Despite these conditions, users rated the system as easy to use and they judged the learning experience as pleasant and enjoyable. Volunteers reported that the mental effort required to operate the device was minimal. Similarly, users indicated that the head-mounted display was comfortable. Unlike other head-mounted displays, such as the Google Glass used in Chaballout’s study [6], no delay between the real and virtual environment was experienced.

Some users noted that ambient light affected the quality of the holograms and made visual instructions difficult to interpret. Users judged the lack of peripheral view, which is known to increase cognitive load [18], as a major limitation. The assembled environment was considered complex enough to be of use, allowing users to focus on the tasks without leaving them disoriented. It is worth noting that trainees found the virtual contents realistic, stating that the system could provide a real benefit as a training tool and help practitioners be more effective.

In this study, we presented preliminary evidence that validated the Holo-BLSD app as a BLS training tool. Additional studies comparing traditional instructor-led training with low and high-fidelity simulation or other models will be useful.

### Limitations

The limitations of the study are the small number of participants, which makes it difficult to draw clear conclusions about the benefit of the proposed technology on learning outcomes. Nevertheless, this study was intended to provide a proof of concept and to measure the feasibility and acceptability of AR technology in a life support simulation. To this end, positive user responses indicate that future studies are warranted.

### Conclusions

AR is in the early stages of application within health care education, but it has enormous potential. In this study, we presented Holo-BLSD, an AR system for BLS training that offers realistic haptic feedback through a manikin and a virtual scenario that can be easily reconfigured to generate different situations, including extreme and dangerous ones. We found the proposed application to be feasible and acceptable as a tool for self-instruction training. The positive outcomes of this preliminary study make this prototype worthy of future testing.

## Appendix

Multimedia Appendix 1 Complete user questionnaire.

Multimedia Appendix 2 User input.

Multimedia Appendix 3 System of output.

Multimedia Appendix 4 Fidelity of simulation.

Multimedia Appendix 5 Immersion.

Multimedia Appendix 6 Likeability.

## Abbreviations

AED: automated external defibrillator
AR: augmented reality
BLS: basic life support
CPR: cardiopulmonary resuscitation
