# Supplementary material for: Augmented Reality Learning Environment for Basic Life Support and Defibrillation Training: Usability Study
Source: J Med Internet Res. 2020 May 12;22(5):e14910. doi: 10.2196/14910 (PMC7251481; doi:10.2196/14910)
Supplement: Multimedia Appendix 1 [file jmir_v22i5e14910_app1.pdf]

Multimedial Appendix n.1 - Complete questionnaire

Evaluation questionnaire

General information

Role ____ Age ___________ Gender _____

How often do you use mobile Augmented Reality devices and applications like the Ikea 2016 Catalogue, PokemonGo, Anatomy 4D, etc.?
Never ___ Few time ___ Once a month ___ Once a week ___ Everyday ___

How often do you use wearable devices for Augmented Reality like Google Glass, Moverio BT-200, Microsoft Hololens, Vuzix M-100?
Never ___ Few time ___ Once a month ___ Once a week ___ Everyday ___

How often do you use interfaces for hand and body gesture recognition like Kinect, Leap Motion, DataGlove, etc.?
Never ___ Few time ___ Once a month ___ Once a week ___ Everyday ___

Have you ever attended a Basic Life Support & Defibrillation (BLSD) course?
Yes ____ No ____

If yes, when did you attend the BLSD course?
____

Mention here the 3 aspects of the system that you felt as more positive
____

Mention here the 3 aspects of the system that you felt as more negative
____

Suggestions for improvement
____

Usability: General features

System Usability Scale (SUS)

	Completely disagree (1) – Completely agree (5)				
	1	2	3	4	5
I think that I would like to use this system frequently					
I found the system unnecessarily complex					
I thought the system was easy to use					
I think that I would need the support of a technical person to be able to use this system					
I found the various functions in this system were well integrated					
I thought there was too much inconsistency in this system					
I would imagine that most people would learn to use this system very quickly					
I found the system very cumbersome to use					
I felt very confident using the system					
I needed to learn a lot of things before I could get going with this system					

ISO 9241-400

	Completely disagree (1) – Completely agree (5))				
	1	2	3	4	5
The HoloLens device is too bulky or too heavy					
The mental effort (concentration) required to operate the device was very high					
The physical effort required to operate the device was very high					
Arm and hands/fingers fatigue was very high					
Eye fatigue was very high					
Head fatigue was very high					
I would be comfortable using the device for long time					

Overall, the device was
Difficult to use ___ Neutral ___ Easy to use ___ Very easy to use ___

Usability: Detailed features

User input

	Completely disagree (1) – Completely agree (5)				
	1	2	3	4	5
I had the right level of control over what I wanted to do					
The effect of my interaction was easy to predict					
The system did not behave as I expected					
I could not always achieve what I wanted the system to do					
I kept making mistakes interacting with the system					
It was easy to realize when my interaction was requested to continue with the task					
It was easy to realize which interaction was requested					
Accurate pointing with gaze was easy to achieve					
Hand interactions were difficult to perform					
Hand interactions were properly recognized by the system					
Voice interactions were properly recognized by the system					
Interaction with the system was fast enough					
The system responded too slowly during interaction					
I would have preferred alternative interaction methods					
I found the input modalities too sensitive					
I found the input modalities ideal for the task to be performed					

Overall, I would rate the user input as
Unsatisfactory ___ Neutral ___ Satisfactory ___ Very satisfactory ___

System output

	Completely disagree (1) – Completely agree (5)				
	1	2	3	4	5
I found the display appropriate for the tasksguardo e dita					
I felt the field-of-view too limited to see the virtual contents and carry out the task					
Screen was not legible because of outdoor ambient light, reflection or glare					
I felt that the display was flickering too much and computer-generated graphics contents were too unstable					
I thought the words and symbols on screen were easy to read					
The quality of the display affected my performance					
I thought that audio instructions provided were easy to understand					

Overall, I would rate the user output system as
Unsatisfactory ___ Neutral ___ Satisfactory ___ Very satisfactory ___

Fidelity of simulation

	Completely disagree (1) – Completely agree (5)				
	1	2	3	4	5
The simulation of the task was too simplified to be of use					
The simulation behaved in a very unusual manner					
I felt disorientated with the virtual environment					
I had the right level of control over what was happening in the virtual environment					
The virtual environment was too simple to be of use					
The quality of computer-generated graphics contents was very realistic					
The quality of computer-generated graphics contents influenced my performance					
I thought that virtual contents were properly aligned with real ones					

Overall, I would rate the fidelity of the simulation as
Unsatisfactory ___ Neutral ___ Satisfactory ___ Very satisfactory ___

Immersion/Presence

	Completely disagree (1) – Completely agree (5)				
	1	2	3	4	5
The sensorial information provided by the Augmented Reality system gave me the impression of being somewhere else (being "there")					
I was really engaged by the situation and I felt part of what was happening in the virtual world (sense of presence)					
The quality of the graphics contents reduced the level of immersion / sense of presence					
The display field-of-view reduced the level of immersion / sense of presence					

Likeability/effectiveness

	Completely disagree (1) – Completely agree (5)				
	1	2	3	4	5
The system is pleasant					
I enjoyed using the system					
I would use this system					
I can see a real benefit in systems like this one					
The system could help to be more effective					
The system could help to be more productive					
The system could make the things I want to accomplish easier to get done					
The system meets my needs					
The system does everything I would expect it to do					
