# Supplementary figures and images for: Augmented Reality Learning Environment for Basic Life Support and Defibrillation Training: Usability Study
Source: J Med Internet Res. 2020 May 12;22(5):e14910. doi: 10.2196/14910 (PMC7251481; doi:10.2196/14910)

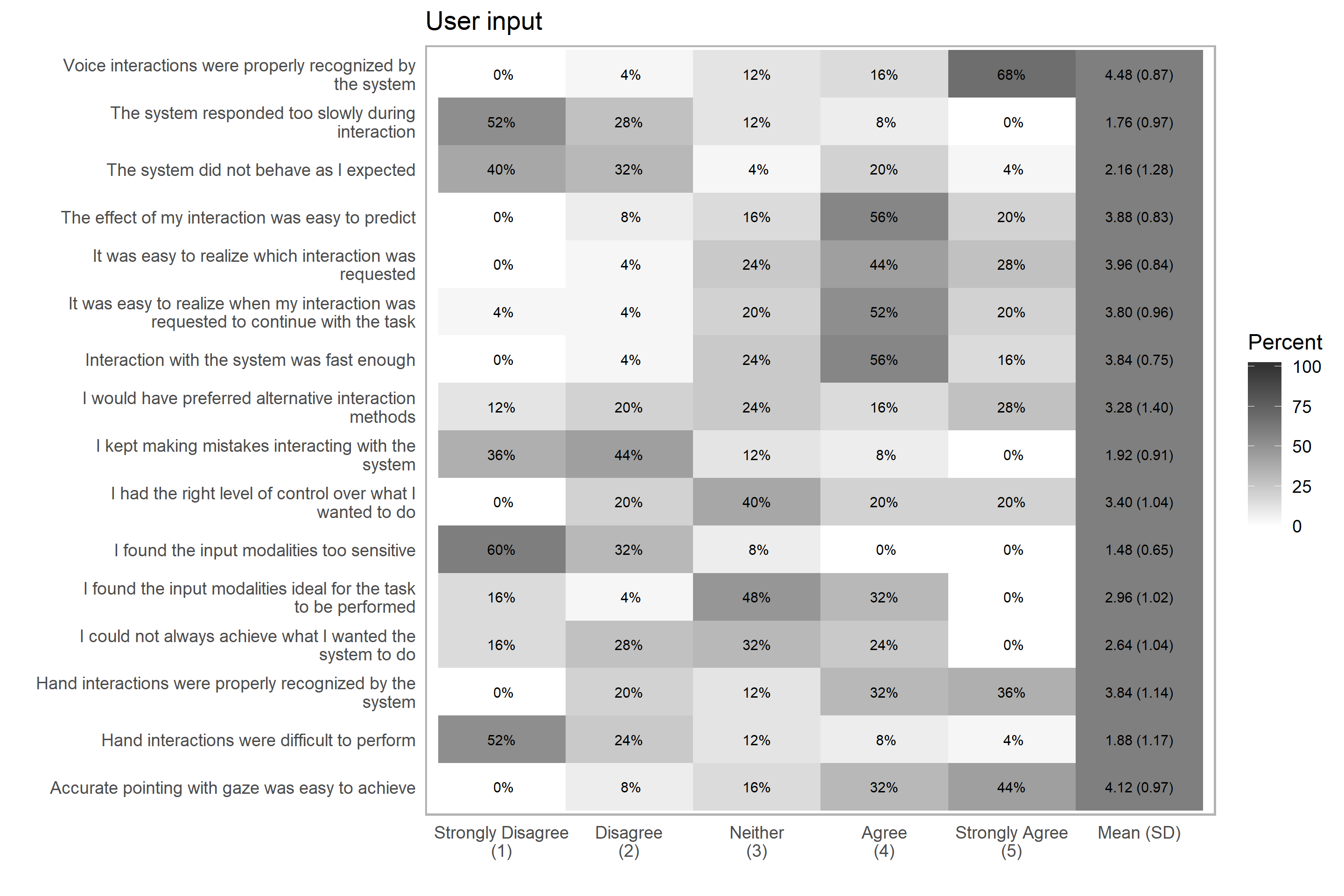

Supplement: Multimedia Appendix 2 [file jmir_v22i5e14910_app2.png]

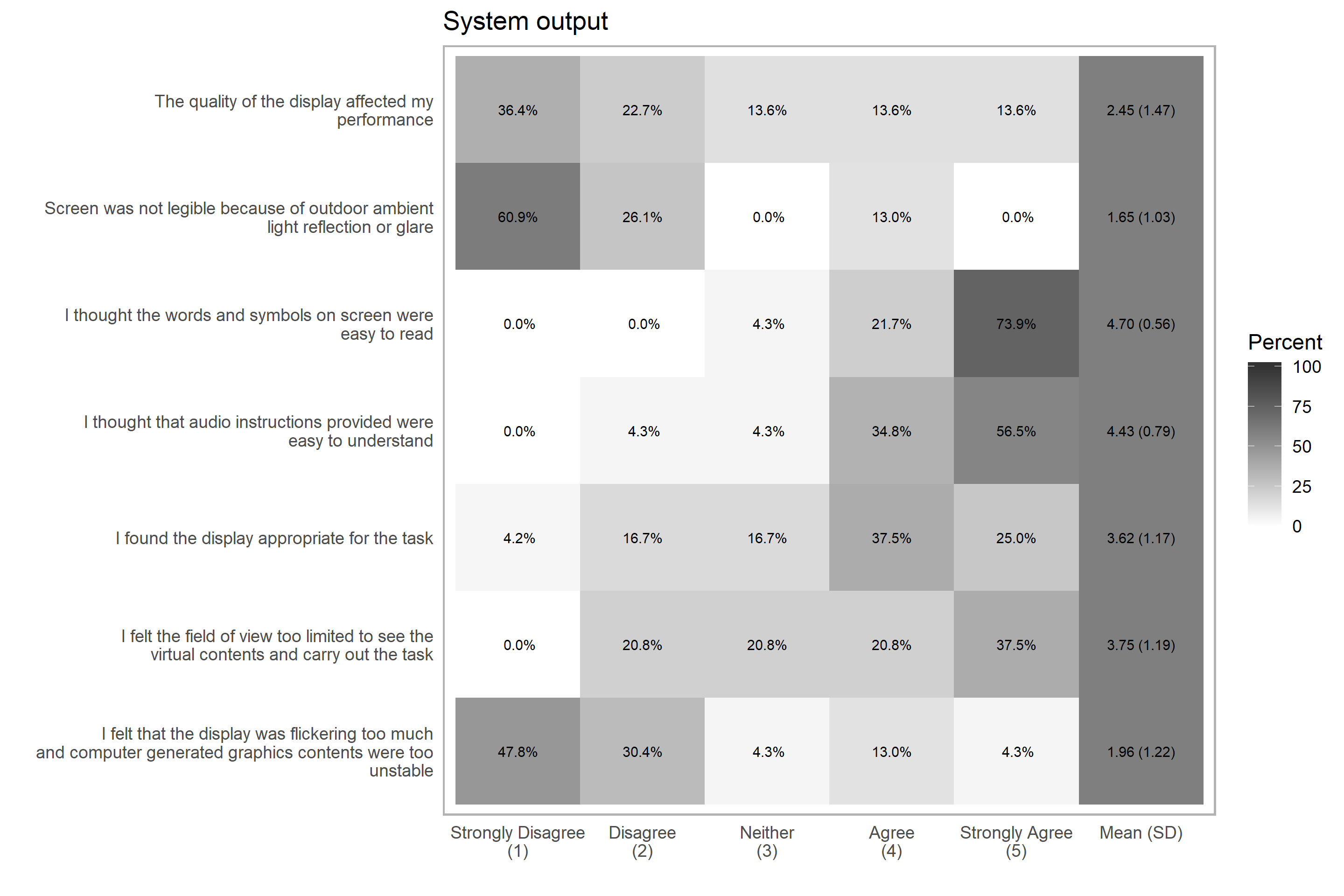

Supplement: Multimedia Appendix 3 [file jmir_v22i5e14910_app3.png]

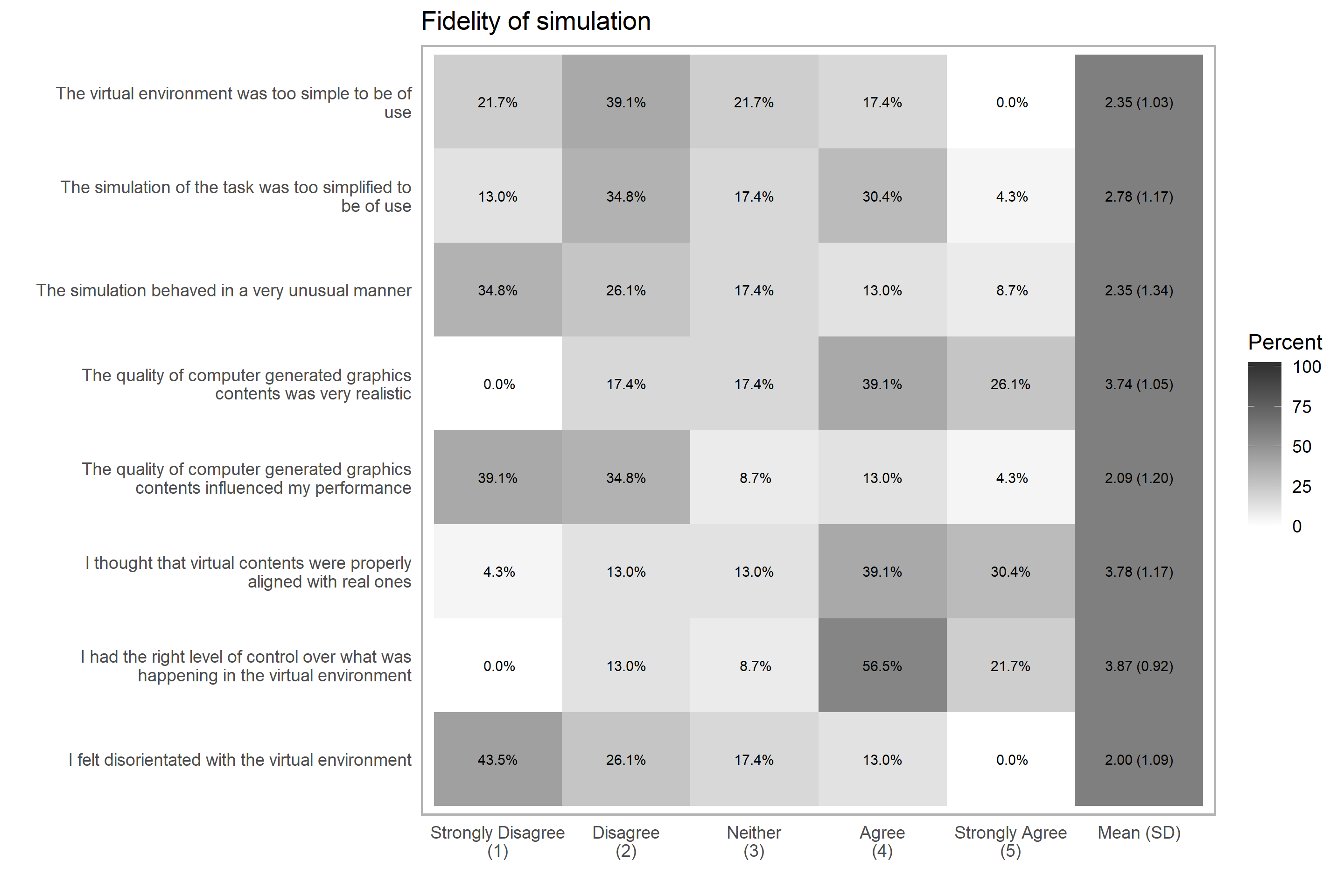

Supplement: Multimedia Appendix 4 [file jmir_v22i5e14910_app4.png]

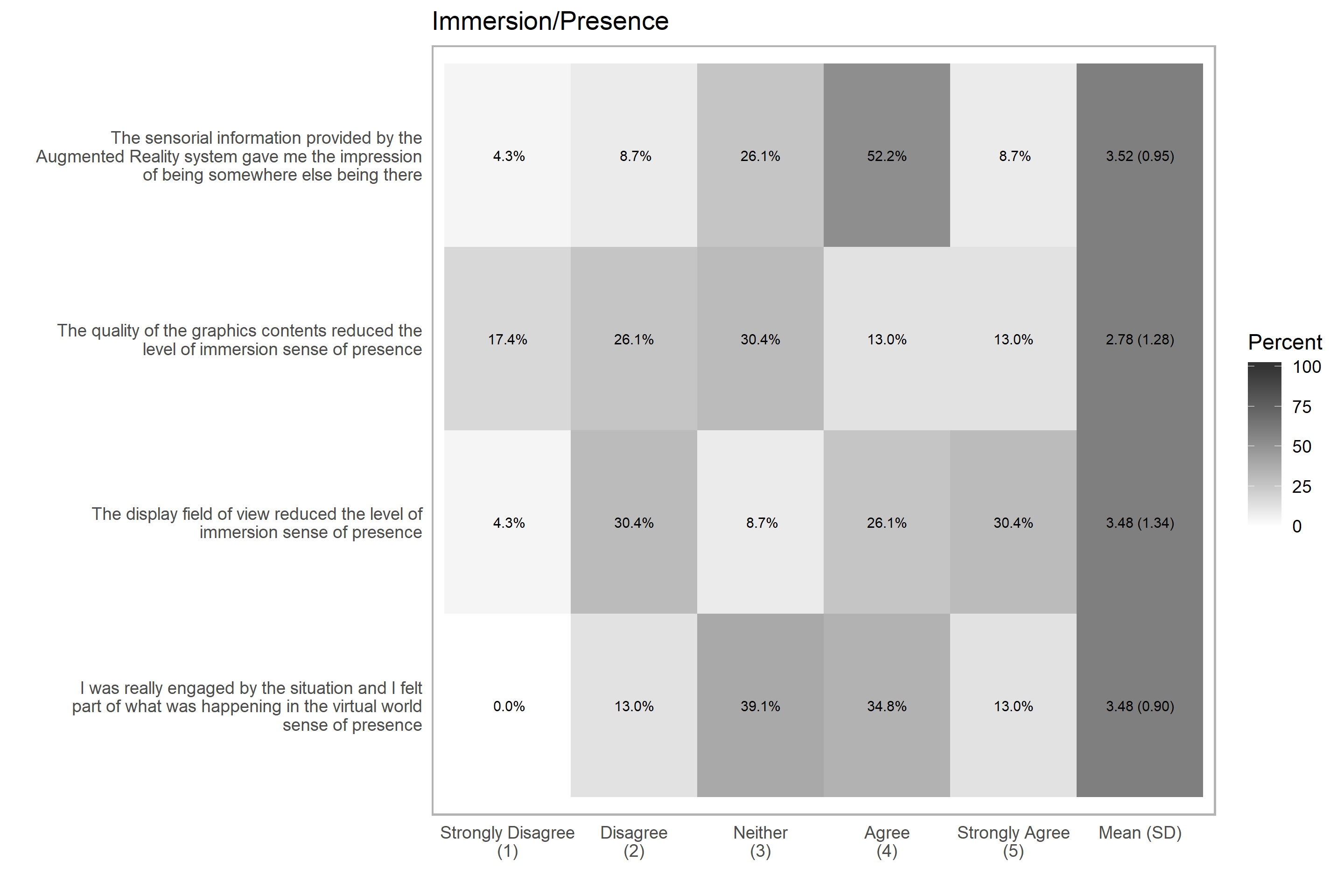

Supplement: Multimedia Appendix 5 [file jmir_v22i5e14910_app5.png]

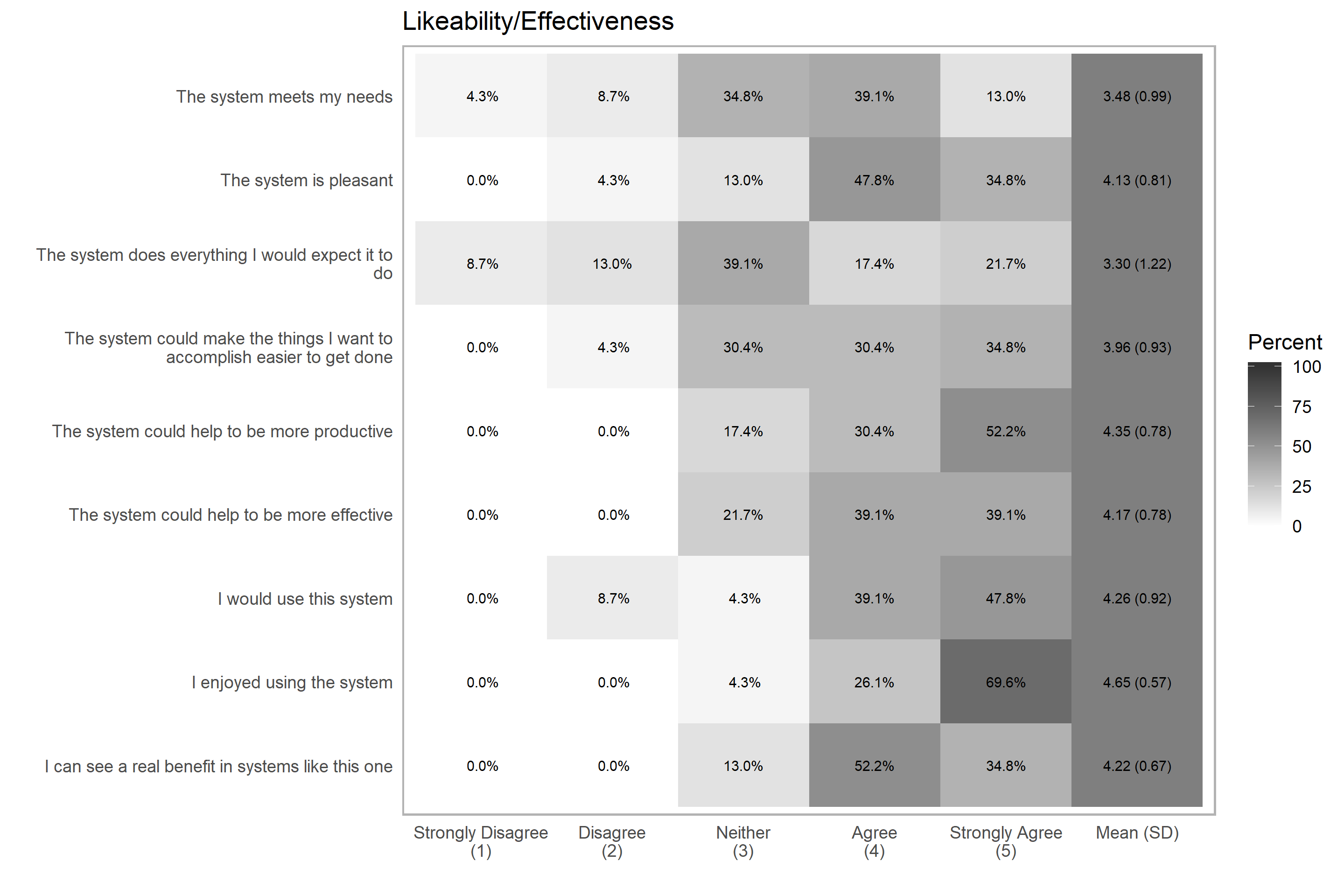

Supplement: Multimedia Appendix 6 [file jmir_v22i5e14910_app6.png]
